# SARS-CoV2 is not just infection but a culprit of donor graft failure post-allogeneic stem cell transplant

**DOI:** 10.46989/001c.121430

**Published:** 2024-07-26

**Authors:** Yoojin Park, Silvia Park, Wichai Chinratanalab, Bipin Savani, Adetola Kassim, Jonathan J Douds, Salyka Sengsayadeth, Tae Kon Kim

**Affiliations:** 1 Duke University; 2 Medicine Vanderbilt University Medical Center; 3 Department of Hematology The Catholic University of Korea; 4 Vanderbilt-Ingram Cancer Center; 5 VA Tennessee Valley Healthcare System; 6 Department of Pathology, Microbiology, and Immunology

**Keywords:** donor graft failure, SARS-CoV2

The patient was a 74-year-old gentleman with intermediate risk acute myeloid leukemia, who achieved first complete remission (CR1) after treatment with venetoclax/decitabine, and underwent a reduced intensity conditioning (RIC) matched unrelated donor (MUD) allogeneic stem cell transplant (allo-SCT). His early post-transplant course had minimal complications. His day +30 bone marrow (BM) exam revealed complete remission and BM chimerism showed nearly 100% donor. Unfortunately, he sustained severe acute respiratory syndrome coronavirus 2 (SARS-CoV2) infection at day +48, requiring inpatient hospitalization and systemic therapy with Nirmatrelvis/ritonavir (Paxlovid) and steroids. Two weeks after his initial diagnosis of SARS-CoV2, his counts were noted to be dropping. Repeat BM biopsy showed hypocellular marrow but chimerism remained at 100% donor (**Figure 1**). The patient required multiple transfusions. His counts improved modestly with systemic steroid, eltrombopag and G-CSF. His day-100 BM exam revealed significant hypocellularity but 100% donor chimerism. His pancytopenia continued to worsen, and his course was complicated by febrile neutropenia and bleeding. Eventually he expired at day +160 due to complications from severe pancytopenia.

**Figure 1.**
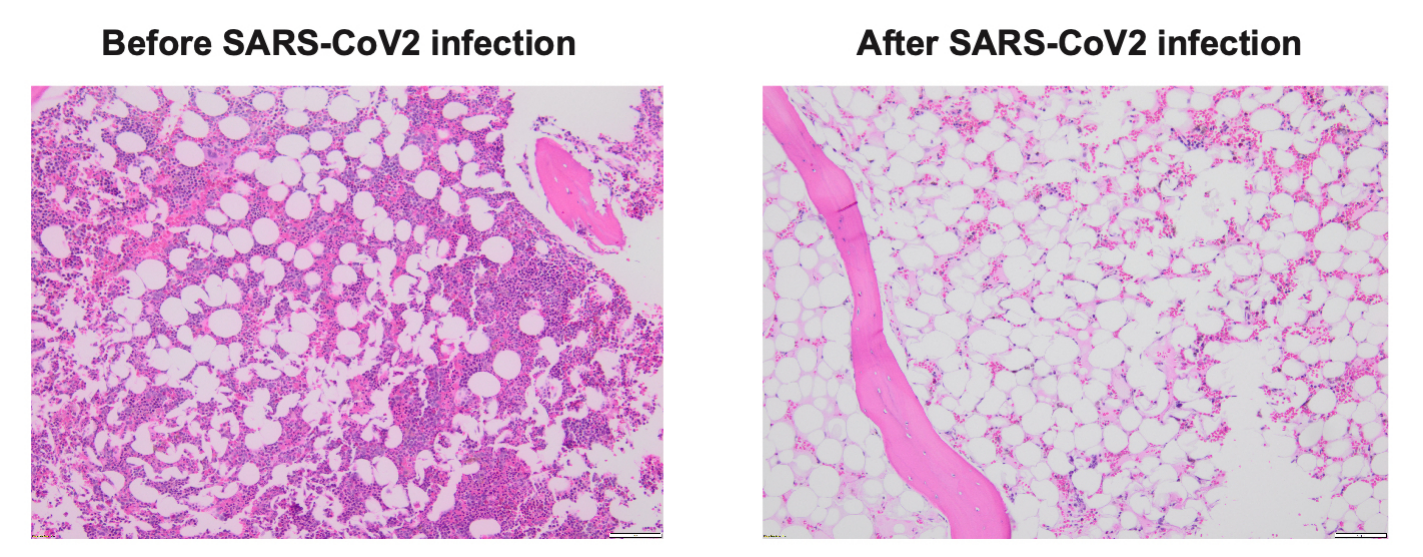
SARS-CoV2 infection suppresses bone marrow post-allogeneic stem cell transplant. Compared with day +30 post-SCT bone marrow, bone marrow biopsy after SARS-CoV2 infection revealed significantly low cellularity.

Recipients of allo-SCT are at high risk for viral infection due to delayed immune recovery and immune-suppressive therapy. One of the most significant causes of opportunistic viral infection post-HSCT is human cytomegalovirus infection (CMV), which is associated with an increased risk of graft failure and mortality.^1,2^ In addition to CMV, there have been reports of either primary or secondary graft failure or poor graft function caused by parainfluenza virus, herpes simplex virus type 1, and parvovirus B19 infection.^3–5^ However, the impact of SARS-CoV2 infection on allo-SCT recipients remains to be elucidated. SARS-CoV2 causes pneumonia that often results in poor clinical outcomes. especially in pre- or early post-engraftment phase.^6^ SARS-CoV2 can also lead to profound cytopenia and even graft failure in patients after allo-SCT.^7,8^ The case we report showed significant pancytopenia associated with hypoplastic marrow following SARS-CoV2 infection. As the patient experienced a decline in hematopoietic function after prior engraftment, but continued to maintain complete chimerism, this could be classified as poor graft function rather than secondary graft failure.^9,10^ Although we could not completely exclude other possibilities responsible for graft dysfunction, such as older age and unrelated donor, we believe that the viral infection, specifically COVID-19 in this case, may substantially contribute to the development of graft dysfunction given its sequential occurrence.

Several studies have reported that SARS-CoV2 induces BM suppression,^11,12^ manifested by pancytopenia, thrombocytopenia alone, or lymphopenia alone.^12,13^ Its etiology remains to be elucidated. SARS-CoV2-induced BM suppression may be associated with thrombosis, such as disseminated intravascular coagulation (DIC). Patients with severe SARS-CoV2 disease frequently develop thrombotic complications due to the hyperinflammatory response caused by the virus.^14^ In addition, hyper-inflammation is caused by excessive neutrophil infiltration and increased formation of neutrophil extracellular traps (NETs).^15^ Further, some metabolic conditions associated with states of chronic inflammation can increase neutrophil predisposition to form NETs.^16^ The components of NETs can also abnormally activate the coagulation pathway and participate in the formation of pathological thrombi that cause BM suppression.^17^

SARS-CoV2 induces the cytokine storm that leads to BM suppression.^18^ Vanderbeke et al (2021) report that neutrophils contribute to disease severity and local tissue damage by amplifying hyper-cytokinemia and forming NETs.^19^ Another cause of inflammation is the increased levels of inflammatory cytokines, including IL-1β, IL-2, IL-6, IL-10, IFN-γ, TNF-α, IFN-γ-inducible protein 10 (IP-10), granulocyte macrophage-colony stimulating factor (GM-CSF), and monocyte chemoattractant protein-1 (MCP-1).^20^ These cytokines correlate with the severity of SARS-CoV2.^21,22^ Specifically, increased IFN-γ results in BM failure by disrupting hematopoietic stem cell (HSC) differentiation and by driving hematopoietic collapse.^23^

SARS-CoV2 may dysregulate hematopoiesis that is associated with BM failure. Lymphoid-primed progenitor cells were dramatically depleted and the accumulation of immature granulocyte-monocyte progenitor cells were found in BM of severe SARS-CoV2 patients.^11^ It was shown that HSCs in these patients were mainly in the G1 phase and prone to apoptosis, with immune activation and anti-viral responses. Along with these findings, there was an up-regulation of transcription factors (such as SPI1, LMO4, ETS2, FLI1, and GATA2) that are important for HSCs or for the differentiation of multipotent progenitors.^11^ Severe SARS-CoV2 patients had a significantly low percentage of HSCs, common myeloid progenitors, and granulocyte-macrophage progenitors compared to non-severe cases.^24^

In addition, COVID-19 may cause hemophagocytic lymphohistiocytosis (HLH), which is associated with BM failure. HLH is an immune-mediated process characterized by an uncontrolled activation and disinhibition of cytotoxic T cells, macrophages, and natural killer (NK) cells.^25^ Recent studies noted high incidence of hemophagocytosis in BM and provided data to support evidence of secondary HLH in SARS-CoV2 patients.^26,27^ The clinical characteristics of SARS-CoV2 resemble secondary HLH, including high cytopenia, thrombocytopenia, and dyspnea.^28^ In HLH, the inability of NK and CD8+ cytotoxic T lymphocytes (CTLs) to provide critical negative feedback in response to an immunologic trigger leads to uncontrolled activation of CTLs and macrophages and initiation of a “cytokine storm”.^25,29,30^ At the initial phase of the severe infectious stage of SARS-CoV2, it is difficult to attribute inflammatory reactions solely to HLH versus SARS-CoV2 infection without other evidence, especially from BM biopsy.^31^ Therefore, conclusive pathophysiology behind post- SARS-CoV2 HLH is yet to be found.^31^

Certain viruses, such as parvovirus B19, may infect HSCs directly, causing changes in BM production. Parvovirus B19 infection can trigger an acute cessation of red blood cell production, leading to transient aplastic crisis, chronic red cell aplasia, hydrops fetalis, or congenital anemia.^32^ In persistent B19 infection, there is generally a decrease or absence of erythroid precursors, with sparing of other BM lineages.^33^ It is not known whether SARS-CoV2 infects HSCs directly.

In conclusion, SARS-CoV-2 infection leading to BM suppression is rare. However, we report a case of BM graft failure associated with SARS-CoV2. It would be difficult to demonstrate the causality of SARS-CoV2 infection with BM suppression in patients. While SARS-CoV2 specific T cells can be assessed using *ex vivo* studies (i.e. tetramer staining for SARS-CoV2 specific T cells, cytokine flow cytometry, multiplex immunoassays),^34^ cross-sectional assessment may limit the causality of SARS-CoA2 infection on BM suppression. Despite this limitation, SARS-CoV2 induced cytokine storm, dysregulated hematopoiesis, and HLH are all possible factors that affect the BM graft and can cause donor graft failure. Patients who receive allo-SCT are more prone to BM graft failure due to SARS-CoV2 infections, and should be carefully monitored for the early identification of BM suppression.

## Authors’ Contribution

Writing – original draft: Yoojin Park (Equal), Tae Kon Kim (Equal). Writing – review & editing: Wichai Chinratanalab (Equal), Bipin Savani (Equal), Adetola Kassim (Equal), Jonathan J Douds (Equal), Salyka Sengsayadeth (Equal), Tae Kon Kim (Equal). Visualization: Jonathan J Douds (Lead). Conceptualization: Salyka Sengsayadeth (Equal), Tae Kon Kim (Equal).

## Competing of Interest

TKK is a consultant for Agenus and Immunobiome but is irrelevant to this project.

## Ethical Conduct Approval – Helsinki – IACUC

(for more information, read https://chi.scholasticahq.com/for-authors)

Not applicable.

## Informed Consent

All authors and institutions have confirmed this manuscript for publication.

## Data Availability Statement

All are available upon reasonable request.
